# Proteomic profiling of neuromas reveals alterations in protein composition and local protein synthesis in hyper-excitable nerves

**DOI:** 10.1186/1744-8069-4-33

**Published:** 2008-08-12

**Authors:** Hong-Lei Huang, Cruz-Miguel Cendan, Carolina Roza, Kenji Okuse, Rainer Cramer, John F Timms, John N Wood

**Affiliations:** 1 Molecular Nociception Group, NPP Department, UCL, Gower Street, London, WC1E 6BT, UK; 2 Dpto. Fisiología Universidad de Alcalá Edificio de Medicina, Campus Universitario28871 Alcalá de Henares, Madrid, Spain; 3 Division of Cell & Molecular Biology Faculty of Natural Sciences Imperial College, London, SW7 2AZ, UK; 4 Department of Chemistry and The BioCentre, University of Reading, PO Box 221, Reading, RG6 6AS, UK; 5 Cancer Proteomics Laboratory, EGA Institute for Women's Health, University College London, Gower Street, London, WC1E 6BT, UK

## Abstract

Neuropathic pain may arise following peripheral nerve injury though the molecular mechanisms associated with this are unclear. We used proteomic profiling to examine changes in protein expression associated with the formation of hyper-excitable neuromas derived from rodent saphenous nerves. A two-dimensional difference gel electrophoresis (2D-DIGE) profiling strategy was employed to examine protein expression changes between developing neuromas and normal nerves in whole tissue lysates. We found around 200 proteins which displayed a >1.75-fold change in expression between neuroma and normal nerve and identified 55 of these proteins using mass spectrometry. We also used immunoblotting to examine the expression of low-abundance ion channels Nav1.3, Nav1.8 and calcium channel α2δ-1 subunit in this model, since they have previously been implicated in neuronal hyperexcitability associated with neuropathic pain. Finally, S^35^methionine in vitro labelling of neuroma and control samples was used to demonstrate local protein synthesis of neuron-specific genes. A number of cytoskeletal proteins, enzymes and proteins associated with oxidative stress were up-regulated in neuromas, whilst overall levels of voltage-gated ion channel proteins were unaffected. We conclude that altered mRNA levels reported in the somata of damaged DRG neurons do not necessarily reflect levels of altered proteins in hyper-excitable damaged nerve endings. An altered repertoire of protein expression, local protein synthesis and topological re-arrangements of ion channels may all play important roles in neuroma hyper-excitability.

## Background

Neuropathic pain is the most problematic of pain conditions. Mechanistic studies using animal models of neuropathic pain have focused on transcriptional profiling of sensory neuron cell bodies. For example, after damage to peripheral nerves, up- regulation of the voltage-gated sodium channel Nav1.3, the calcium channel α2δ1 subunit, the B1 bradykinin and capsaicin TRPV1 receptors were observed, whilst other transcripts encoding, for example, the Nav1.8 sodium channel, B2 bradykinin receptor and μ-opioid receptor were down-regulated [1-4]. All of these data were acquired from comparison of dorsal root ganglion (DRG) tissues of control animals and those in which neuropathic pain was induced by ligation and dissection of peripheral nerve. However, the molecular changes in terms of altered protein components of injured nerves which underlie neuropathic pain have not been studied, mainly because of the difficulty in obtaining sufficient tissue material for proteomic profiling.

In the present research, we ligated and transected the saphenous nerves of mice and rats to generate neuromas, which we have previously shown to be hyper-excitable and mechanically sensitised. When peripheral sensory nerves are damaged, the axon distal to the section degenerates, whereas the proximal portion sprouts, and if regeneration towards the innervation target is impeded, a neuroma is formed. After neuroma formation, spontaneous activity appears to be generated at trigger zones either at the regenerating nerve ending (neuroma) or in the DRG cell bodies [5,6]. Regenerating nerve endings in a neuroma are also sensitised to mechanical forces locally [7,8]. Neuromas are attractive models of nerve injury, and have also been suggested to exhibit many of the properties of peripheral nerve terminals [8].

To try to better understand the molecular mechanisms underlying neuropathic pain at the protein level, we have compared protein expression during neuroma formation with normal peripheral nerve using an unbiased 2D-difference gel electrophoresis (2D-DIGE) and mass spectrometry (MS)-based profiling approach. A large number of differentially expressed protein isoforms were identified and some of these further validated by immunoblotting with specific antibodies. Since large hydrophobic transmembrane proteins do not resolve well on 2D gels, we extended this profiling approach by using immunoblotting to examine the abundance of several voltage-gated ion channels (Nav1.3, Nav1.8 and calcium channel α2δ-1 subunit) which have previously been implicated in the response to nerve damage [1-4]. Finally, we used in vitro S^35 ^methionine labelling to test the hypothesis that local protein synthesis occurs in neuromas after nerve injury and independently of gene expression.

## Methods

### Neuroma formation

The method used for neuroma generation was adapted from that described in rats by Rivera et al. [8]. Ten C57BL/6 mice were used to generate neuromas; all mice were matched by age (8 weeks) and weight (30–35 g). Under deep anaesthesia with halothane (2–4% in pure O_2_) and with sterile precautions, the saphenous nerve was exposed at the level of the mid-thigh, dissected free and tightly ligated with 8-0 silk. The nerve was cut distal to the ligature and the cut end inserted into a 5 mm long tube (0.45 mm internal diameter) to prevent lateral innervation of surrounding tissue. The tube was tied in place with the same piece of silk so that the cut end of the nerve was ~2 mm from the distal end of the tube. The distal end of the tube was left open and ~5 mm of the distal nerve stump was excised to prevent innervation. The incision was closed in layers. Neuromas were made in both saphenous nerves. The animals were housed in groups of two to four and inspected daily for infections or abnormal behaviour. The mice were given *ad libitum *access to water and food. After 3 days, 1 week, 2 weeks and 3 weeks, the silicone tube was removed, neuroma were cleared of connective and fatty tissues and dissected for 2D-DIGE experiments under a dissecting microscope. Four neuroma samples were generated from 2 mice in each time point after transection. At the same time, 4 control samples were generated from two control C57BL/6 mice without operation. Similar experiments were carried out on Sprague-Dawley rats. 32 rats were equally distributed in 4 groups (3 day, 1 week, 2 week and 3 week), one neuroma and one sham-operation control were generated from each rat. All rats were matched by age (3–4 months old) and weight (200–250 g). All animal care and experimental processes were performed in accordance with the United Kingdom Home Office Act 1986.

### Chemicals

Protease-inhibitor tablets, Tris-base, urea, thiourea, ammonium persulfate, ammonium bicarbonate, iodoacetamide (IAA) and 3- [(3-cholamidopropyl)-dimethylammonio]-1-propanesulfonate) (CHAPS) were from Sigma (Steinheim, Germany). Protein assay kit was from Pierce (Rockford, USA). Immobilized pH gradient IPG strips and Pharmalyte were from GE Healthcare (Chalfont St. Giles, UK). Dithiothreitol (DTT) was from Melford Laboratories (Ipswich, UK). Trypsin (modified, sequence grade, porcine) was from Promega (Madison, WI, USA). 2,5-dihydroxybenzoic acid was from Bruker Daltonics (Leipzig, Germany). Ammonium sulphate and ortho-phosphoric acid (85%) were from Fluka (Steinheim, Germany). Methanol, ethanol and Coomassie brilliant blue G250 were from BDH (Lutterworth, UK). TFA was from Fisher (Loughborough, UK). NHS-Cy2 was purchased from GE Healthcare. NHS-Cy3/Cy5 were synthesized 'in-house' and stored as described [9].

### Sample preparation

Pooled neuroma or control nerve samples in 1.5 ml centrifuge tubes were frozen in liquid nitrogen and crushed into powder with a plastic homogenization pestle. Samples were lysed in 2D-DIGE lysis buffer (2 M thiourea, 8 M urea, 4% (w/v) CHAPS, 10 mM Tris-HCl, pH 8.3) and quantified with a Bradford assay using BSA as a standard.

### Fluorescence labelling with Cy-Dyes

Neuroma and control samples were quantified and labelled with NHS-Cy2, -Cy3 and -Cy5 as previously described [10]. Briefly, 50 μg of protein taken from pooled neuroma or control samples was minimally labelled with 400 pmol of Cy3 or Cy5 in triplicate. An equal pool of all samples was also prepared and labelled with Cy2 (also at 8 pmol/μg protein) as a standard run on all gels to aid in spot matching and cross-gel quantitative analysis. Labelling was performed on ice in the dark for 1 hour and reactions were quenched with a 20-fold molar excess of free lysine to dye for 10 minute, and then DTT added to 65 mM. In addition, two preparative samples were made using 300 μg of protein from control saphenous nerve labelled with Cy3 or Cy5. Labelled samples were mixed appropriately and carrier Ampholines/Pharmalyte (pH 3–10; 50:50(v/v)) added to a final concentration of 2%.

### Two-dimensional gel electrophoresis (2-DE)

2-DE was performed as described [11-13] using 24 cm pH 3–10 NL IPG strips, and 12% SDS-PAGE gels bonded to low-fluorescence glass plates. Briefly, Cy3 and Cy5 labelled control, 3 day, 1 week, 2 week or 3 week neuroma samples and one Cy2 labelled internal standard sample were mixed appropriately and separated by first dimension isoelectric focusing and second dimension SDS-PAGE. Samples were run as technical triplicates with two preparative gels loaded with 300 μg of protein. Gels were run in Ettan 12 gel tanks at 2.2 mA per gel at 16°C until the dye front had run off the bottom. All steps were carried out in a dedicated clean room.

### Image acquisition and biological variance analysis

Gels were scanned between glass plates using a Typhoon™ 9400 variable mode imager and ImageQuant software. The photomultiplier tube voltage was adjusted for each dye channel (Cy2, Cy3 and Cy5) for preliminary low-resolution scans to give maximum pixel values within 5–10% for each channel and below saturation, prior to the acquisition of 100 μm high-resolution images. Images were cropped and analysed using DeCyder™ V5.0. Comparisons of test spot volumes with corresponding standard spot volume were made and values were averaged across triplicates for each experimental condition. Statistical analysis was then performed to pick spots matching across all images, displaying a ≥1.75 average-fold increase or decrease in abundance between neuroma and control and with p values < 0.05 (Student's *t*-test). A 1.75-fold threshold was chosen to limit the substantial number of significant protein changes detected that would require MS-based protein identification, but without missing potentially interesting low-fold changes. It is important to note that only significant changes (p < 0.05) in protein expression were targeted for MS-based identification.

### In-gel digestion and protein identification by mass spectrometry (MS)

Post-stained colloidal Coomassie blue (CCB) G-250 images were matched with corresponding fluorescent images using DyCyder software. Pick lists of coordinates were then created for spots of interest relative to a pair of reference markers fixed to the glass plates at casting. Spots were excised using an Ettan spot picker (GE Healthcare). In-gel tryptic digestion was performed as described [9,10], and extracted peptides were resuspended in 6 μL of water. For peptide mass fingerprinting, 0.5 μL of each tryptic digest was mixed with 1 μL of matrix (saturated aqueous solution of 2,5-dihydroxybenzoic acid) and spotted onto a sample target plate and dried. Matrix-assisted laser desorption/ionization time-of-flight (MALDI-TOF) mass spectra were acquired using an externally calibrated Ultraflex TOF/TOF mass spectrometer (Bruker Daltonics) in the reflector mode. After internal calibration using trypsin autolysis peaks, prominent peaks in the mass range *m/z *700–4000 were used to generate a peptide mass fingerprint which was searched against the updated mouse IPI database using Mascot version 2.1.3 (Matrix Sciences, London, UK). Identifications were accepted when a minimum of six peptide masses matched to a particular protein (mass error of ± 50 ppm allowing 1 missed cleavage), sequence coverage was >25%, MOWSE scores were higher than the threshold value (p = 0.05) and the predicted protein mass agreed with the gel-based mass. Nano-HPLC electrospray ionization (ESI) collision-induced dissociation (CID) MS/MS was performed on a Q-TOF mass spectrometer (Waters, Manchester, UK), coupled to an Ultimate system LC (Dionex) with a PepMap C18 75 μm inner diameter column at a flow rate of 300 nL/min. Spectra were processed using MassLynx software (Waters) and submitted to Mascot database search routines. Positive identifications were made when at least two peptide sequences matched an entry and MOWSE scores were above the significance threshold value (p = 0.05)

### Immunoblotting

Since hydrophobic integral membrane proteins are difficult to resolve on 2D-gels, and previous data suggested that altered ion channel expression was correlated with pain [14-18], the expression of sodium channel Nav1.3, Nav1.8 and calcium channel α2δ-1 subunit were measured by immunoblotting. Briefly, extracts from rat neuromas separated by 1D-SDS-PAGE were electro-blotted onto Hybond-C Extra membrane (GE Healthcare). Membranes were blocked for 1 h with 5% w/v BSA in TBS (50 mM Tris HCl pH 8, 150 mM NaCl) plus 0.1% Tween-20 (TBS-T). Membranes were incubated for at least 2 h in primary antibody in TBS-T, washed in TBS-T (3 × 10 min) and probed with the appropriate horseradish peroxidase-coupled secondary antibody (GE Healthcare) for 1 h. After further washes in TBS-T (3 × 10 min), immunoprobed proteins were visualised using ECL (Perkin-Elmer Life Sciences, Waltham, MA, USA). Films were scanned on a Bio-Rad GS800 Densitometer. Polyclonal antibodies used were anti-Nav1.8 (1:2500 dilution; generated in-house), anti-Nav1.3 (1:2500 dilution; Sigma), anti-voltage calcium channel α2δ-1 subunit (1:500 dilution; a kind gift from Professor A Dolphin, UCL), anti-protein DJ-1 (1:1000 dilution; Santa Cruz Biotechnology, Calne, UK), anti-vimentin (1:1500 dilution; DAKO, Ely, UK) and anti-Na^+^/K^+^ATPase (1:1000 dilution; Santa Cruz Biotechnology). Monoclonal antibodies used were anti-actin beta (1:2000 dilution; Sigma) and anti-peroxiredoxin 2 (1:5000 dilution; Lab Frontier, Seoul, South Korea).

### S^35 ^methionine *in vitro *labelling

Mouse neuroma samples were generated as described. One week old neuromas from two mice were dissociated and washed 3 times with cold PBS then transferred to methionine-free DMEM medium for 30 min. S^35 ^methionine (GE Healthcare) was added to a final concentration of 100 mCi/mL and samples were incubated in a 5% CO_2 _incubator at 37°C overnight. S^35^-labelled samples were washed 3 times in cold PBS, homogenised and lysed in 2D lysis buffer. The S^35^-labelled sample (50 μg total protein) was mixed with 200 μg of intact sciatic nerve and separated by 2-DE as described above. Gels were stained with CCB, dried, scanned by densitometry and then placed in a cassette with a phosphorimager screen (Kodak, Hemel Hempstead, UK) for 5 days. Screens were scanned with a Molecular Imager FX (Bio-Rad Laboratories, Hemel Hempstead, UK) and radio-labelled spots were picked from dried gels and identified by MS.

## Results and Discussion

### Protein expression profiling of control and neuroma samples

**Figure 1:**
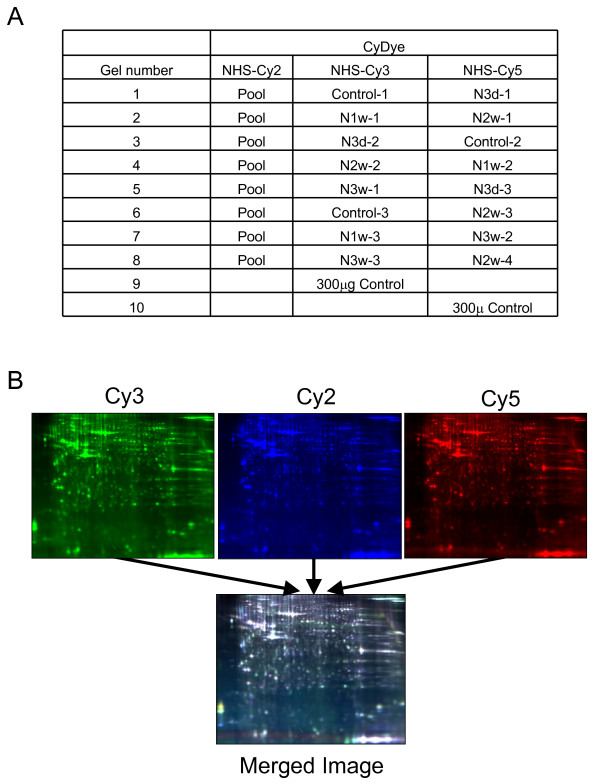
**Strategy for comparing neuroma and nerve cell protein composition.** (A) Dye-labelling strategy for 2D-DIGE experiment profiling of mouse neuroma and control saphenous nerves. Control-1 to Control-3 indicates triplicate control samples. N3d-1 to N3d-3 indicates triplicate 3 day neuroma samples. N1w-1 to N1w-3 indicates triplicate 1 week neuroma samples. N2w-1 to N2w-4 indicates quadruplicate 2 week neuroma samples. N3w-1 to N3w-3 indicates triplicate 3 week neuroma samples. Additional preparatory gels were run with a higher loading of control sample. (B) Representative example of a 2D-DIGE analytical gel: 50 μg of control sample was labelled with NHS-Cy3 shown in green, 50 μg of neuroma sample was labelled with NHS-Cy5 shown in red and 50 μg of pooled internal standard (equal amounts of control and neuroma sample) was labelled with NHS-Cy2 shown in blue. The three Cy-dye labelled samples co-separate on one gel. Images were acquired on a Typhoon 9400 mutli-wavelength scanner. Around 1800 protein spots were detected on each gel. Below is a merged image of the three Cy-dye labelled images.

We used both proteomic and immunoblotting approaches to catalogue altered protein expression in hyper-excitable neuromas derived from the saphenous nerve. 2D gels lose many of the highly insoluble receptors and channels present at low abundance in cell membranes during the isoelectric focussing phase, so we also carried out immunoblotting to examine some proteins previously implicated in neuropathic pain. A 2D-DIGE analysis was first performed comparing 3 day, 1 week, 2 week and 3 week old neuromas with control uninjured saphenous nerve. Four samples from two animals were pooled from each condition, generating 160–210 μg of total protein and samples were analysed as technical triplicates using an internal standard on each gel consisting of an equal pool of all samples to aid in spot matching and quantification (Figure 1).

**Figure 2:**
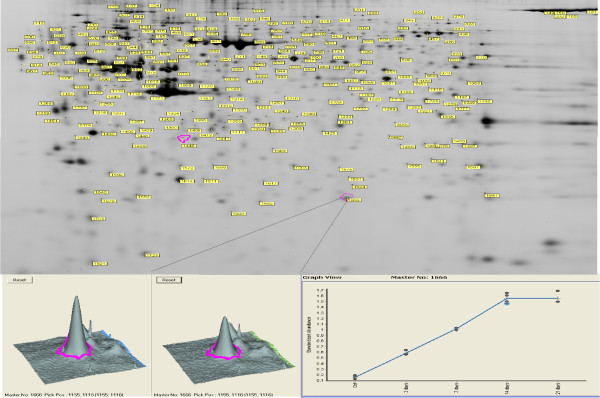
**2D-DIGE DeCyder BVA (Biological Variation Analysis) showing a representative gel image with labelled sample of control and neuroma.** Changes in protein expression were compared between 4 time-points (3 day, 1 week, 2 week and 3 week neuromas) and controls after normalisation with a pooled internal standard. The master gel image shows the locations of differentially expressed proteins (≥1.75 change in abundance; p < 0.05; n = 3). The lower part of the figure shows an enlarged region of the gel and 3-D view of up-regulated spot 1666 as an example (lower left). Spot no 1666 (protein DJ-1) is shown in detail as displaying increased expression during neuroma formation (lower right).

Approximately 1800 protein features were resolved on the gels and compared using DeCyder image analysis software (Figure 2). A Student's t-test was performed for every matched spot set, comparing the average and standard deviation of protein abundance across each condition. There were around 200 spots that were 1.75-fold differentially expressed (p ≤ 0.05) between the neuroma and control groups (see Table 1). For example, there were 218 spots detected with a ≥1.75-fold change between 2 and 3 week neuromas and control with 138 increased in neuroma and 80 decreased in neuroma versus control.

**Table 1:** Numbers of protein spots differentially expressed between 3 day, 1 week, 2 week, 3 week and 2 week plus 3 week neuromas and control saphenous nerves.

	3 day neuroma/control	1 week neuroma/control	2 week neuroma/control	3 week neuroma/control	2 and 3 week neuroma/control
Total number of differentially expressed spots (≥1.75-fold)	224	248	254	182	218
Increased	137	141	153	125	138
Decreased	87	109	101	57	80

Spots displaying a ≥1.75-fold increase or decrease in expression with a Student T-test P value of ≤ 0.05 (n = 3/4) and matching across all 8 analytical gels were included.

### Identification of differentially expressed proteins

Differentially expressed spots detected in analytical gels were matched and picked from preparative gels run at the same time (loaded with 300 μg protein from control nerves). Combined MALDI-TOF MS and Q-TOF LC-MS/MS analysis identified 55 protein spots with a high degree of confidence. The identification of each protein is set out in Table 2 with MS search results, the description of average fold-changes in abundance between 2 and 3 week neuroma versus control nerve, the t-test *P *value and a description of known molecular function. According to function, the identified proteins could be classified into several main groups:

**Table 2:** Differentially expressed proteins identified from 2D-DIGE profiling of neuroma and control saphenous nerve samples.

**Spot no**.	**Protein name**	**Accession no**.	**% Cov**	**MOWSE score**	**Est. mass**	**Est. pI**	**Pred. mass**	**Pred. pI**	**Av. ratio (2 and 3 week neuroma vs. control)**	***P*-value (t-test)**	**Function**
**Structural proteins**											

1171	Beta-actin	IPI00110850	8	107/35	46073	5.1	42051	5.29	-1.84	3.00E-05	Major cytoskeletal protein involved in cell motility
1173	Beta-actin	IPI00110850	33	190/35	40780	5.2	42051	5.29	-1.9	0.00095	Major cytoskeletal protein involved in cell motility
1234	Macrophage capping protein	IPI00136906	9	154/35	39216	6.7	39241	6.73	2.64	2.90E-09	Calcium-sensitive protein which reversibly blocks barbed ends of actin filaments.
553	Plastin-2	IPI00118892	11	103/35	69112	5.1	70019	4.96	1.75	1.10E-05	Actin-bundling protein
638	Plastin-2	IPI00118892	4	36/35	66167	5.2	70019	4.96	4.03	5.00E-07	Actin-bundling protein
857	Tubulin alpha-1	IPI00110753	10	66/35	57828	5.0	50788	4.94	-2.59	3.60E-05	Major microtubule protein
902	Tubulin, alpha 6	IPI00403810	44	100/64*	56122	5.0	49877	4.96	-3.29	4.00E-06	Major microtubule protein
912	Tubulin alpha 2	IPI00117348	23	122/64*	55894	5.0	50120	4.94	-3.77	4.40E-07	Major microtubule protein
966	Tubulin, beta 5	IPI00117352	16	708/35	54467	5,.0	49671	4.52	-2.41	3.00E-05	Major microtubule protein
973	Tubulin beta-2C	IPI00169463	7	365/35	53804	4.6	49832	4.52	2.47	1.70E-06	Major microtubule protein
196	Neurofilament triplet M	IPI00323800	24	100/64*	96041	4.8	95941	4.76	-4.69	8.20E-07	Maintenance of neuronal caliber
636	Neurofilament triplet L	IPI00169702	49	630/35	66257	4.7	61378	4.3	-5.93	3.80E-06	Maintenance of neuronal caliber
939	Vimentin	IPI00227299	25	113/35	55515	5.2	53557	4.77	1.76	0.00039	Class-III intermediate filament protein
997	Vimentin	IPI00227299	21	191/35	52933	4.8	53557	5.06	2.19	7.20E-07	Class-III intermediate filament protein
1008	Vimentin	IPI00227299	28	195/35	52431	5.1	53557	5.06	3.05	1.30E-07	Class-III intermediate filament protein
1108	Vimentin	IPI00227299	27	349/35	48584	4.7	53557	5.06	-2.67	0.00018	Class-III intermediate filament protein
1112	Vimentin	IPI00227299	20	180/35	48123	5.0	53557	5.06	5.11	8.90E-09	Class-III intermediate filament protein
839	Peripherin (isoform 5g)	IPI00129527	9	100/35	58302	5.5	54268	5.22	-9.58	2.20E-06	Class-III neuronal intermediate filament protein
858	Peripherin (isoform 5g)	IPI00129527	15	363/35	58064	5.4	54268	5.22	-3.44	5.50E-07	Class-III neuronal intermediate filament protein

**Metabolic enzymes**											

734	Aspartyl-tRNA synthetase	IPI00122743	21	70/64*	62153	5.0	57117	6.06	1.84	0.00046	Protein biosynthesis
907	Aspartyl-tRNA synthetase	IPI00122743	28	66/64*	56122	6.5	57117	6.06	2.87	2.30E-06	Protein biosynthesis
817	D-3-phosphoglycerate dehydrogenase	IPI00225961	8	111/35	59666	6.2	56455	6.5	2.5	3.90E-06	Glycolytic enzyme
1031	Alpha-enolase	IPI00462072	33	526/35	51162	6.8	47010	6.37	1.78	0.00056	Glycolytic enzyme and other functions
1038	Alpha-enolase	IPI00462072	10	40/35	50954	6.9	47010	6.37	2.06	1.20E-05	Glycolytic enzyme and other functions
1039	Alpha-enolase	IPI00462072	56	369/35	51093	7.0	47010	6.37	1.9	3.10E-07	Glycolytic enzyme and other functions
1049	Gamma-enolase	IPI00331704	15	90/35	50677	5.8	47166	4.73	2.08	3.80E-06	Has neurotrophic and neuroprotective properties
807	Pyruvate kinase M2	IPI00407130	4	55/35	59910	7.4	58004	7.52	-1.85	5.20E-06	Glycolytic enzyme
965	Cytosolic nonspecific dipeptidase	IPI00315879	6	72/35	54467	4.9	52768	5.38	-2.62	3.70E-05	Non-specific dipeptidase activity
1359	Lactate dehydrogenase B chain	IPI00229510	22	128/35	37311	5.8	36442	5.7	-2.79	3.60E-06	Glycolytic enzyme
1567	Carbonic anhydrase 3	IPI00221890	62	307/35	26265	7.5	29235	6.89	-2.15	8.40E-06	Reversible hydration of carbon dioxide
1570	Carbonic anhydrase 3	IPI00221890	31	413/35	26229	7.6	29235	6.89	-2.54	2.70E-05	Reversible hydration of carbon dioxide
770	Pyruvate kinase M2	IPI00407130	10	148/35	60897	7.5	58536	7.18	2.19	9.40E-05	Glycolytic enzyme
801	Pyruvate kinase M2	IPI00407130	18	218/35	59910	7.3	58004	7.52	-2.21	3.30E-06	Glycolytic enzyme
958	ATP synthase subunit beta, mitochondrial precursor	IPI00468481	35	266/35	54616	4.9	56300	5.19	-4.82	7.80E-07	Produces ATP from ADP
1011	ATP synthase subunit beta, mitochondrial precursor	IPI00468481	6	787/35	52005	5.0	56300	5.19	2.08	3.30E-06	Produces ATP from ADP
1143	Creatine kinase B-type	IPI00136703	30	337/35	47023	5.5	42714	5.42	-1.89	0.00036	Transfer of phosphate between ATP and phosphogens
1189	Creatine kinase B-type	IPI00136703	32	244/35	45388	4.8	42714	5.42	4.71	5.00E-08	Transfer of phosphate between ATP and phosphogens

**Redox regulation**											

1666	Protein DJ-1	IPI00117264	25	68/35	20021	6.3	20021	6.32	9.91	1.60E-09	Positive regulator of androgen receptor-dependent transcription, protects neurons against oxidative stress and cell death
1618	Peroxiredoxin 1	IPI00121788	43	75/64*	23048	7.3	22177	8.26	1.93	0.00022	Involves in redox regulation of the cell
1647	Peroxiredoxin 1	IPI00121788	46	211/35	22117	8.3	22177	8.26	3.52	1.40E-06	Involves in redox regulation of the cell
1678	Peroxiredoxin 2	IPI00117910	30	88/35	19551	5.1	21648	5.2	1.79	0.002	Involves in redox regulation of the cell

**Cell signalling**											

789	Rab GDP dissociation inhibitor alpha	IPI00323179	24	290/35	60731	4.9	50522	4.7	-2.01	1.00E-05	Regulates the GDP/GTP exchange reaction of Rab
1430	Annexin A1	IPI00230395	20	87/35	36121	4.9	35752	4.83	1.78	1.30E-05	Promotes membrane fusion and is involved in exocytosis
1454	Annexin A4	IPI00353727	44	136/35	32168	5.4	36121	5.43	2.4	1.60E-08	Promotes membrane fusion and is involved in exocytosis

**Protein folding/chaperone function**											

768	Protein disulfide isomerase associated A3	IPI00230108	8	103/35	60897	5.9	57099	5.88	2.03	2.60E-05	Protein folding and electron transport
820	Protein disulfide isomerase associated A3	IPI00230108	35	525/35	57099	5.9	57099	5.88	2.51	9.00E-08	Protein folding and electron transport
824	Protein disulfide isomerase associated A3	IPI00230108	5	67/35	59504	5.9	57099	5.88	1.91	0.00063	Protein folding and electron transport
825	Prolyl 4-hydroxylase, beta polypeptide	IPI00122815	30	357/35	59343	4.8	57422	4.77	2.24	7.10E-07	Catalyzes the rearrangement of -S-S- bonds in proteins
778	Calreticulin precursor	IPI00123639	12	159/35	60237	4.4	47995	4.09	2.67	8.10E-07	Protein folding
862	T-complex protein 1 subunit beta	IPI00320217	6	62/35	57907	6.5	57347	6.37	2.22	0.00031	Molecular chaperone, assist the folding of proteins upon ATP hydrolysis

**Others**											

1572	Calpain small subunit 1	IPI00130992	9	85/35	25980	5.3	28464	5.34	4.83	4.30E-05	Catalyzes limited proteolysis of substrates involved in cytoskeletal remodelling and signal transduction.
1283	Guanine nucleotide-binding protein G(o) subunit alpha 2	IPI00115546	4	86/35	41603	5.8	39906	5.69	-2.3	8.50E-05	Modulator or transducer in various transmembrane signalling systems
1415	Mimecan precursor	IPI00120848	11	153/35	34668	5.9	34012	5.52	-1.8	0.0032	Induces bone formation in conjunction with TGF-beta-1 or TGF-beta-2
1821	Gamma-synuclein	IPI00271440	65	247/35	13152	4.7	13160	4.68	-1.87	0.00066	Plays a role in neurofilament network integrity
1598	Ubiquitin C-terminal hydrolase isozyme L1	IPI00313962	45	279/35	24437	5.2	24839	5.14	-2	3.70E-05	Ubiquitin-protein hydrolase

Proteins were identified using a combination of LC-ESI-MS/MS peptide sequencing and MALDI-TOF MS peptide mass fingerprinting. The International Protein Index (IPI) accession number, the percentage coverage of the protein sequence where matching peptides were found (% Cov), the MOWSE score and threshold score (at *P *= 0.05) from Mascot searches, the estimated (from 2D gels) and predicted (from database) molecular mass and pI, the average fold-change in spot volume between the 2 and 3 week neuroma and control samples, Student's t-test *P *value and known molecular function is given for each protein. Proteins are divided into six broad functional groups. * MOWSE score and significance threshold from Mascot searches using MALDI-TOF peptide mass fingerprinting data.

#### Structural proteins involved in cytoskeletal organization

These included several tubulin isoforms, major components of the microtubule network. Tubulins α1, α2, α6 and tubulin β5 were expressed at lower levels, whereas tubulin β2C was more highly expressed (2.74-fold) in the neuroma versus control samples. Intermediate filament proteins were also represented. Neurofilament triplet M and L proteins were down-regulated in neuromas (-4.69-fold and -5.93-fold, respectively), as was the class III neuronal intermediate filament protein peripherin (isoform 5 g). Five spots were found to contain isoforms of vimentin. Four of these were more highly expressed in neuroma and one was reduced in expression, suggesting differential post-translational modification of isoforms. Vimentin is a class-III intermediate filament protein and is one of the most prominent phosphoproteins found in cell types of mesenchymal origin. Vimentin phosphorylation is enhanced during cell division, at which time vimentin filaments are significantly reorganised [19,20]. In the present study, 4 of the 5 vimentin isoforms identified were overexpressed in the neuroma samples, suggesting enhanced filament reorganisation after peripheral nerve injury. Vimentin has also been shown to play an important role in the spatial translocation of activated MAP kinase (pERK) in injured nerves [21]. Here, local synthesis of vimentin at the axonal lesion site (confirmed by S^35 ^labelling; see below) was proposed to allow linkage of pERK to a retrogradely transported complex via a direct interaction of vimentin with importin beta. After travelling from the axon to the DRG, pERK dissociates from vimentin and is then available to phosphorylate downstream targets in the cell body or the nucleus, for example, transcription factors to control the proliferation or apoptosis of the neuron [21]. In several animal models of neuropathic pain, nerve injury-induced phosphorylation of ERK occurs early and is long-lasting [22]. pERK is sequentially activated in neurons by spinal nerve ligation and contributes to mechanical allodynia in neuropathic pain models [23].

Components of the actin cytoskeleton also showed changes. Expression of two β-actin isoforms was increased, whilst an α-actin isoform was reduced. Actin binding proteins, usually associated with immune system cells, were also altered in expression. The actin-bundling protein plastin-2 (two spots) was up-regulated, as was macrophage capping protein (2.64 fold). Macrophage capping protein (CapG) is a calcium-sensitive actin-modulating protein that binds to the plus ends of actin monomers or filaments. It can promote the assembly of monomers into filaments, but not sever pre-formed filaments [24]. CapG is expressed broadly, although its up-regulation may correspond to invasion of the neuroma by immune cells. The multiple changes in cytoskeletal proteins of all filament types may also be related to the complex neurite outgrowth that occurs in developing neuromas.

#### Metabolic enzymes

Two proteins which play a central role in energy transduction were differentially expressed. Two protein spots identified as ATP synthase beta subunits showed a 2.08-fold up-regulation and 4.82-fold down-regulation in neuroma, respectively, suggesting that changes in post-translational modifications may occur during formation of the neuroma. Similarly, two protein spots were found to contain creatine kinase B-type, and showed a 4.71-fold up-regulation, and 1.89-fold down-regulation in neuroma versus control. Other metabolic or protein synthesis enzymes were identified as aspartyl-tRNA synthetase (two spots), D-3-phosphoglycerate dehydrogenase, prolyl 4-hydroxylase beta polypeptide, alpha-enolase (three spots) and gamma-enolase, all of which were more highly expressed in neuroma, whereas pyruvate kinase isozyme M2, cytosolic non-specific dipeptidase, lactate dehydrogenase B chain and carbonic anhydrase 3 (two spots) were expressed at a lower level.

#### Redox proteins

Several proteins involved in the elimination of peroxides and redox regulation were identified. Both peroxiredoxin-1 (two spots) and peroxiredoxin-2 were up-regulated (1.93-, 3.52- and 1.79-fold, respectively) in neuromas. Peroxiredoxin 1 and 2 are a family of ubiquitous proteins that function as antioxidant cytoprotective proteins in all metazoan and protozoan organisms. In general, they utilise electrons from thioredoxin or other sulphydryl donors to reduce cellular peroxides, including hydrogen peroxide [25]. Peroxiredoxins may participate in the signalling cascades of growth factors and tumour necrosis factor-alpha by regulating the intracellular concentration of H_2_O_2 _[26]. The up-regulation of peroxiredoxin 1 and 2 suggests that oxidative insult is part of the nerve injury process, and that they play a role in protecting proteins and lipids against oxidative damage.

Another putative redox regulator, protein DJ-1/PARK7, showed a linear increase with time during neuroma formation, increasing 9.91-fold by 3 weeks (Table 2 and Figure 2). The DJ-1 protein also affects cell survival by modulating PTEN/PI3K/AKT-regulated signalling cascades and alters p53 activity [27,28]. Recently, DJ-1 was associated with Parkinson's disease and the protection of neurons from oxidative stress [28]. Under oxidising conditions, DJ-1 is activated by the modification of cysteine residues to cysteine-sulfinic and cysteine-sulfonic acids to exert its protective effects [29,30]. Other redox-sensitive proteins were also identified including protein disulfide isomerase-associated 3, a multi-functional enzyme mainly located in the endoplasmic reticulum which catalyses the disruption and formation of disulfide bonds. Tanaka et al reported that protein disulfide isomerase is up-regulated in response to hypoxia/brain ischemia in glial cells [29], whilst increased protein disulfide isomerase expression has been shown to be neuroprotective in the rat hippocampal CA1 region [31].

#### Protein folding/chaperones

Several proteins that function as molecular chaperones were identified. For example, prolyl 4-hydroxylase beta polypeptide was 4.77-fold up-regulated in neuroma. Three spots were identified as protein disulfide isomerase A3 and all were up-regulated (see above). Calreticulin precursor and T-complex protein 1 subunit beta were also more highly expressed in neuroma. Calreticulin functions as a molecular calcium binding chaperone promoting folding, oligomeric assembly and quality control in the ER via the calreticulin/calnexin cycle. T-complex protein 1 subunit beta is a molecular chaperone, assisting in the folding of proteins such as actin and tubulin.

#### Other proteins

Annexins A1 and A4 were up-regulated 1.78-fold and 2.4-fold in the neuroma samples, respectively. The annexins are a family of structurally-related proteins whose common property is calcium-dependent phospholipid binding and which promote membrane fusion and exocytosis. Annexin A1 plays a role in many diverse cellular functions, such as membrane aggregation [32], inhibition of arachidonic acid mobilisation and phagocytosis [33]. Rab GDP dissociation inhibitor-alpha (RabGDIα), a protein involved in the regulation of vesicle-mediated cellular transport was 2.01-fold down-regulated. RabGDIα regulates GDP/GTP exchange of most Rab proteins by inhibiting the dissociation of GDP. The Rab family of small GTPases are essential regulators of intracellular membrane sorting and membrane trafficking [34]. Ishizaki et al demonstrated that loss of RabGDIα in mice leads to neural hyper-excitability and the mice become prone to epileptic seizures [35]. The down-regulation of RabGDIα could thus play a role in hyper-excitability of neuromas. Calpain small subunit 1 displayed increased expression in the neuromas, whilst guanine nucleotide-binding protein Go subunit alpha 2, ubiquitin carboxyl-terminal hydrolase isozyme L1 (UCH-L1), gamma-synuclein, mimecan precursor were all down-regulated (Table 2). Of these, UCH-L1 is neuron-specific and is expressed throughout the brain and in Lewy bodies, the pathological biomarker of Parkinson's disease. UCH-L1 plays an important role in ubiquitin-dependent proteolysis by recycling polymeric chains of ubiquitin to monomeric ubiquitin. Ubiquitin is activated, conjugated and ligated to damaged proteins for proteasomal degradation. Disruption of the ubiquitin proteasomal system and resultant cytoplasmic aggregation of alpha-synuclein has been implicated as a potential cause of Parkinson's disease [36].

### Validation by immunoblotting

**Figure 3:**
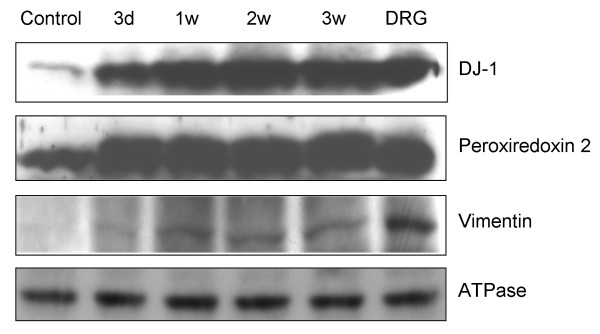
**Western blotting confirmation of 2D-DIGE results for candidate proteins DJ-1, peroxiredoxin 2 and vimentin. **Na^+^/K^+^-ATPase detection was used as a loading control. Neuroma and control sample was taken from rat. Thirty μg of total protein from dorsal root ganglia (DRG) dissected from normal mice were loaded as a positive control.

Differential expression of several of the identified proteins was validated by immunoblotting, although samples from rats were used, due to insufficient material being obtained from the mice. The expression of protein DJ-1 increased with time of neuroma formation as shown from the 2D-DIGE/MS profiling of mouse samples (Figure 3). Similarly, the increased expression of peroxiredoxin 2 during neuroma formation was also confirmed, as was vimentin expression. Importantly, the immunoblotting results of rat neuromas were consistent with the 2D-DIGE analysis of mouse samples, showing that the observed changes can be translated across species and are likely to be functionally relevant in neuroma formation.

### Ion channel protein expression level in rat neuroma

**Figure 4:**
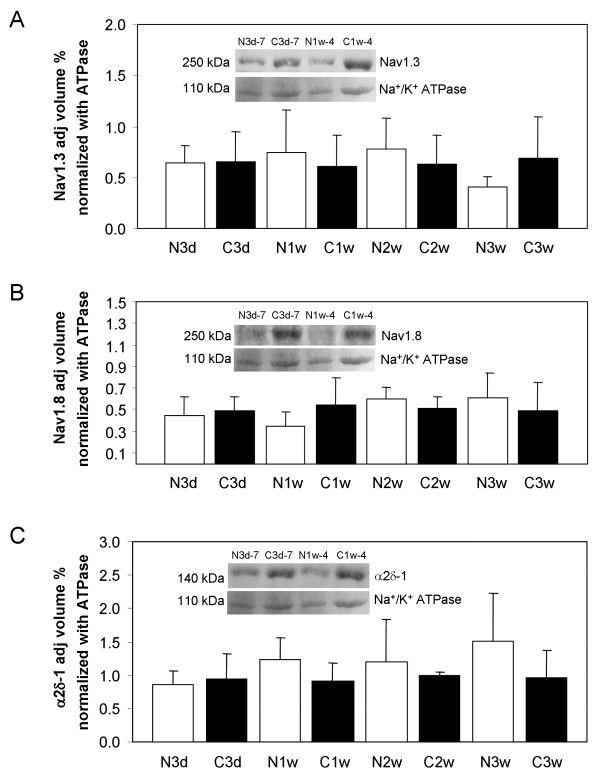
**Graphs showing expression of sodium channel Nav1.3 (A), Nav1.8 (B) and calcium channel α2δ-1 subunit (C) in rat neuromas and control nerves as determined by western blotting.** Columns represent the mean adjusted band volume (%) ± SD after normalisation with the Na+/K+ ATPase loading control. N3d, N1w, N2w and N3w are 3 day, 1 week, 2 week and 3 week neuroma groups. C3d, C1w, C2w and C3w are 3 day, 1 week, 2 week and 3 week normal nerve control group. Data was combined from the analysis of individual samples in three blotting experiments. Representative blots are shown within each graph for animals 7 and 4. No significant changes were detected for any of the proteins between neuroma and control groups.

Various ion channels have been implicated in the pain response. However, due to their very hydrophobic nature and large masses, these proteins cannot be readily extracted from samples or resolved by 2-DE. Thus, sodium channels Nav 1.3 and Nav 1.8 and calcium channel α2δ-1 subunit expression were evaluated in individual rat neuromas and control nerves from the same animals by immunoblotting samples which had been lysed directly in boiling SDS-PAGE sample buffer. Loading was then normalised against Na^+^/K^+^-ATPase (Figure 4). The expression of Nav 1.3 appeared to be the same between 3 day neuroma and control, slightly increased in 1 week and 2 week neuromas and slightly decreased in 3 week neuromas. However, these differences were not significant when the loading was normalised to Na^+^/K^+^-ATPase (Figure 4A). Similarly, although neuroma-specific changes in expression were suggested for Nav 1.8 and calcium channel α2δ-1 subunit, these changes were not consistent when the protein loading was normalised against Na^+^/K^+^-ATPase (Figure 4B–C). We conclude that there are no significant changes in the expression of these proteins during neuroma formation.

### Examination of local protein synthesis in neuromas

**Figure 5:**
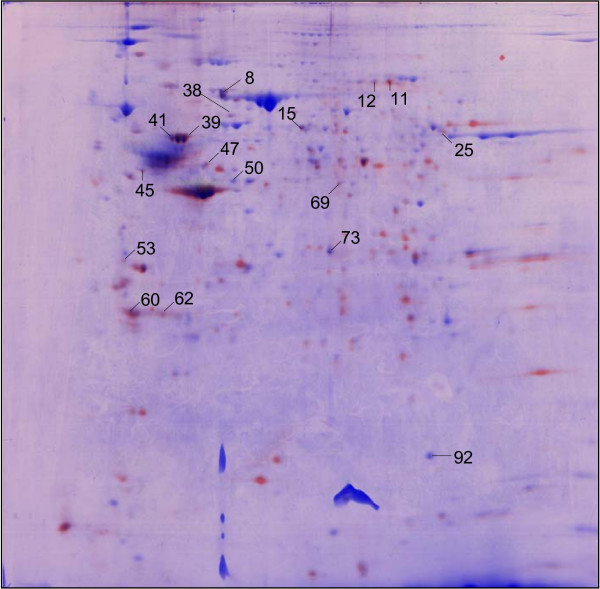
**Local protein synthesis in neuromas using in vitro S^35 ^methionine labelling.** The figure shows merged images of a CCB-stained neuroma sample (blue), and autoradiographs of S^35^-methionine-labelled neuroma (red) and S^35^-methionine-labelled control sample (green).

Neuromas and control saphenous nerves were incubated *in vitro *with S^35 ^methionine to detect *de novo *protein synthesis in the absence of the cell body/DRG. 2-DE was then used to separate and compare protein synthesis between the neuroma and control samples. Interestingly, most of the S^35^-labelled protein spots detected were found in the neuroma sample and only a few were present in both sample types (Figure 5). S^35^-labelled and colloidal Coomassie blue-stained spots were picked and digested for identification by MS. Vimentin, moesin, gamma-actin and neuron-specific protein neurofilament-L were among the proteins identified (Table 3). This demonstrates that gene-expression-independent translation of neuron-specific proteins occurs in neuromas. The presence of mRNA encoding a variety of proteins in DRG sensory neurons has been demonstrated by Willis et al [37]. In addition, protein changes in the central nervous system have been measured that are related to peripheral nerve damage [38]. Zheng et al provided evidence that DRG axons can synthesise proteins after crush [39], and these experiments confirm these findings. It is striking that the neuroma, defined as a useful model of the nerve terminal shows much higher levels of protein synthesis than isolated saphenous nerve. This is consistent with a significant role for local protein synthesis in regulation of terminal excitability in normal nerve endings.

**Table 3:** Locally synthesized proteins identified after S^35 ^methionine labelling of neuromas and control saphenous nerves.

**Spot no**	**Protein name**	**Accession no**.	**MOWSE score**	**Est. mass**	**Est. pI**	**% Cov**
53	Annexin A5	gi|74142393	172	35787	4.83	56
39	Vimentin	gi|2078001	127	51590	4.96	52
45	Actin, gamma 1 pro-peptide	gi|4501887	86	42108	5.31	33
11	Moesin	gi|70778915	68	67839	6.22	35
60	Tyrosine 3-monooxygenase/tryptophan 5-monooxygenase activation protein, zeta polypeptide, isoform C (14-3-3 zeta)	gi|148676868	61	29240	4.71	35
41	Vimentin	gi|31982755	57	53712	5.06	22
15	Protein disulfide isomerase associated 3	gi|112293264	52	57099	5.88	28
92	mTERF domain-containing protein 1, mitochondrial precursor	gi|81901619	46	47385	6.18	16
25	Unnamed protein product	gi|74210074	45	30222	9.34	26
38	Heat shock protein 1	gi|31981679	45	61089	5.67	37
12	Protein phosphatase 2, regulatory subunit B, delta isoform	gi|148685891	42	33815	6.18	9
50	Creatine kinase	gi|10946574	41	42971	5.4	24
47	Neurofilament-L	gi|200038	36	61542	4.63	11
62	Unnamed protein product	gi|26352624	40	15849	11.26	7
69	ORM1-like protein 1	gi|81174966	41	17401	9.56	37
73	S100 calcium binding protein G	gi|14789635	41	9150	4.69	57
8	Annexin A6	gi|31981302	42	76294	5.34	22

All proteins were identified by LC-ESI-MS/MS peptide sequencing. NCBI gene identifier (gi|) number, MOWSE score from Mascot searches using LC-MS/MS data, estimated molecular mass and pI, and the percentage coverage of the protein sequence where matching peptides were found are given for each protein.

## Conclusion

We have found changes in the expression and post-translational modification of components of all types of filamentous structures, actin, tubulin and intermediate filaments in mouse neuromas. In addition a number of enzymes associated with oxidative stress and protein chaperones are up-regulated. We accept that the sample number used and the pooling strategy adopted for the 2D-DIGE profiling is not ideal and that there may be considerable animal to animal variation which has been masked. However, immunoblotting of lysates from a larger number of rat neuromas (8 in each pool) with control nerves from the same animals, showed a similar pattern of expression as observed in the mouse model, at least for three of the candidate proteins tested. This supports the notion that the expression changes are indeed consistent from animal to animal as well as across species. Surprisingly, there seemed to be little change in the expression of immuno-reactive sodium channels Nav1.3, Nav1.8 and calcium channel α2δ-1 subunit in neuromas, whilst the mRNA levels encoding these proteins are dramatically altered in the cell bodies of damaged sensory neurons [3]. Local protein synthesis of neuronal-specific proteins such as neurofilament-L chain occurred at higher levels in the neuromas than in normal nerve, suggesting by analogy that local protein synthesis in nerve terminals may occur at a higher level than in nerve trunks. These studies show both the power and limitations of current proteomic technology in following the altered levels of expression of very low abundance membrane-associated insoluble proteins that are key elements in determining neuronal excitability. Whilst a number of cytoplasmic enzymes associated with neuronal damage and death in other neurodegenerative states have been catalogued, the molecular basis of hyper-excitability is likely to reflect the altered topology of voltage-gated channel expression which can be best addressed by antibody studies. Unfortunately, few mono-specific antibodies to ion channels are commercially available, making this approach technically difficult.

Interestingly, a number of markers associated with the characteristic nerve damage observed in Parkinson's disease have been identified in neuromas (e.g. protein DJ-1), suggesting that these proteins are not causally linked to nerve damage in particular pathologies, but are up-regulated as a consequence of nerve damage. RabGDIα down-regulation is known to result in enhanced neuronal excitability, and its disruption may contribute to neuroma excitability changes. However, few of the other catalogued proteins have an obvious role in contributing to ectopic firing. Combining the current approach with immunocytochemical studies of channel distribution should provide a better insight into the underlying mechanisms that result in neuroma hyper-excitability.

## Abbreviations

2D-DIGE: two-dimensional differential gel electrophoresis; Cy2: 3-(4-carboxymethyl) phenylmethyl-3'-ethyloxacarbocyanine halide; Cy3: 1-(5-carboxypentyl)-1'-propylindocarbocyanine halide; Cy5, 1-(5-carboxypentyl)-1'-methylindocarbocyanine halide; BVA: biological variation analysis; MS: mass spectrometry; PMF: peptide mass fingerprinting; MALDI: matrix-assisted laser desorption/ionization; TOF: time-of-flight; LC: liquid chromatography; MS/MS: tandem mass spectrometry; CCB: colloidal Coomassie blue; AmBic: ammonium bicarbonate; Nav 1.8: Voltage gated sodium channel 1.8; RabGDI: Rab GDP dissociation inhibitor alpha; UCH-L1: Ubiquitin carboxyl-terminal hydrolase isozyme L1.

## Competing interests

The authors declare that they have no competing interests.

## Authors' contributions

HLH, CMC and CR carried out the experiments, HLH, JFT and JW designed experiments and wrote the manuscript. KOk and RC were co-applicants for the grant funding to support this study, and helped to write the paper. We confirm that all authors read and approved the final manuscript.
